# Hydroxychloroquine as an Immunomodulatory and Antithrombotic Treatment in Antiphospholipid Syndrome

**DOI:** 10.3390/ijms24021331

**Published:** 2023-01-10

**Authors:** Deepa J. Arachchillage, Mike Laffan, Charis Pericleous

**Affiliations:** 1 Centre for Haematology, Department of Immunology and Inflammation, Imperial College London, London W12 0NN, UK; 2 Department of Haematology, Imperial College Healthcare NHS Trust, London W12 0HS, UK; 3 National Heart and Lung Institute, Imperial College London, London W12 0NN, UK

**Keywords:** antiphospholipid syndrome, thrombosis, inflammation, pregnancy complications, hydroxychloroquine

## Abstract

Antiphospholipid syndrome (APS) is an acquired highly prothrombotic disorder in which thrombo-inflammatory antiphospholipid antibodies (aPL) cause thrombosis via multiple mechanisms, including endothelial damage and activation. Obstetric complications in APS are caused by placental thrombosis, inflammation and complement activation. Anticoagulation is poorly effective in some patients especially those with triple positive aPL who are at ~30% risk of thrombosis recurrence within 10 years. Increasing therapeutic anticoagulation intensity may be beneficial but leads to excess bleeding with serious complications, such as intracerebral haemorrhage. Nonetheless, anticoagulation is still the mainstay of treatment despite the autoimmune nature of APS. The antimalarial immunomodulatory drug hydroxychloroquine (HCQ) has been used for many years for the treatment of inflammatory rheumatic diseases. HCQ has complex pleiotropic mechanisms of action upon multiple cell types. The proposed biological processes that HCQ regulates support the hypothesis that it may be a successful adjunctive treatment in the prevention of recurrent thrombosis and pregnancy complications.

## 1. Introduction

Antiphospholipid syndrome (APS) is the paradigm example of an autoantibody-mediated prothrombotic disorder, resulting in a high frequency of thrombosis and pregnancy complications. Unlike autoimmune vasculitis where inflammation is the hallmark of disease progression, inflammation is not the predominant feature of APS. Instead, the presence of inflammation may act as a “second hit” in the process of developing thrombosis or pregnancy complications in an individual with antiphospholipid antibodies (aPL) [1]. Thrombosis in APS can be venous, arterial, microvascular or a combination of events occurring in any organ or tissue. Deep vein thrombosis is the most common venous thrombotic event, although thrombosis at unusual sites including cerebral venous sinus thrombosis, portal vein thrombosis and retinal vein thrombosis is a presenting feature. Stroke is the most common arterial thrombotic event and around one-third of strokes <50 years of age may be due to APS [1]. Pregnancy complications include recurrent miscarriages and late pregnancy complications such as foetal loss due to placental insufficiency. The diagnosis of APS requires the presence of clinical events (either thrombosis, pregnancy complication or both) with persistently positive aPL (detection on ≥two consecutive occasions at least 12 weeks apart) [2]. Laboratory diagnostic criteria aPL tests measure lupus anticoagulant (LA), IgG/IgM anticardiolipin (aCL) and IgG/IgM anti-β_2_-glycoprotein I (anti-β_2_GPI) antibodies.

In addition to the standardised clinical criteria for APS diagnosis (Sydney criteria) [2], several other clinical features are frequently associated with APS, collectively known as non-criteria features. These include heart valve disease, livedo reticularis, thrombocytopenia, nephropathy and alveolar haemorrhage which may present an association with thrombosis and/or pregnancy morbidity or as isolated features with persistently positive aPL. APS is a relatively rare disease with an estimated UK incidence of 1 in 2000 [3] but it has major clinical significance. APS can present in isolation (primary APS) or in association with other autoimmune diseases, mainly systemic lupus erythematosus (SLE) or rheumatoid arthritis (RA) (secondary APS). A study of 1000 patients with SLE over 10 years demonstrated that, of the 7% overall mortality, 26.5% of those deaths were due to thrombotic APS [4]. Patients with triple-positive aPL (i.e., presence of LA, aCL and anti-β_2_GPI) are at the highest risk of developing first and recurrent events of thrombosis [5].

Besides aCL and anti-β_2_GPI, autoantibodies against various other targets are present in APS patients, which may variably contribute to the pathogenesis of the disorder. Of these, anti-phosphatidylserine/prothrombin antibodies (anti-PS/PT) that target the complex of anionic phospholipid phosphatidylserine (PS) and the procoagulant plasma protein prothrombin (PT) have been shown to be involved in pathogenesis and thrombotic risk prediction [6,7]. This target is analogous to the complex of cardiolipin with β_2_GPI. Recent evidence suggests that LA positivity, determined by functional clotting assays, is likely due to the presence of anti-PS/PT rather than aCL/anti-β_2_GPI [7]. Several studies have shown an enhanced performance of anti-PS/PT compared with antibodies against prothrombin alone (aPT) in APS [8,9].

aPL target multiple cell types and particularly endothelial cells (EC) to cause thrombosis [1]. The endothelium, forming the internal wall of blood vessels, naturally presents an antithrombotic surface to the flowing blood, expressing and secreting factors that modulate vascular contractility, coagulation, fibrinolysis and platelet adhesion. Inflammation reverses this phenotype, leading to the upregulation of procoagulant/antifibrinolytic factors such as the tissue factor (TF), von Willebrand factor (VWF) and plasminogen activator inhibitor-I (PAI-I) and downregulation of anticoagulant/fibrinolytic factors such as thrombomodulin (TM), the tissue factor pathway inhibitor (TFPI), endothelial protein C receptor (EPCR), tissue–plasminogen activator (t-PA) and nitric oxide (NO), creating an environment favouring thrombosis [10]. Additionally, inflammation upregulates cell adhesion molecules (e.g., VCAM, ICAM and selectins), promoting endothelial interaction with blood cells, including platelets and leukocytes. Largely via effects on the vasculature, inflammation plays a critical role in the pathogenesis of thrombosis in APS [11]. The binding of aPL to EC upregulates TF expression and promotes leukocyte adhesion, cytokine secretion and PGE2 synthesis. aPL interfere with naturally occurring anticoagulant pathways, including protein C and TFPI pathways, either by producing antibodies against these anticoagulants or indirectly, for example, via the TM pathway [12]. aPL also inhibits fibrinolysis through various mechanisms, including the downregulation of tPA via antibodies against tPA, disruption of the TM pathway or upregulation of PAI-1. In addition, aPL binds platelets through phospholipid-binding proteins leading to platelet aggregation. All these mechanisms contribute to a prothrombotic state, regarded as the first hit in developing thrombosis. Notably, thrombotic events occur only intermittently and not all individuals with aPL developing thrombosis, underlining the importance of a ‘second hit’ or ‘trigger’ to precipitate thrombosis [1]. A second hit such as inflammation, infection or genetic susceptibility for developing thrombosis tips the balance, and combined with activation of the complement, leads to thrombosis [12].

Although APS is an autoantibody-mediated disease, anticoagulation with vitamin K antagonists (VKA, primarily warfarin) rather than immunomodulation remains the mainstay of management. A major concern in APS is arterial thrombosis, which, unlike atherosclerosis, mainly affects young patients [13] and is driven by pathogenic mechanisms unrelated to conventional vascular risk factors and plaque rupture. For primary and secondary prevention of ischaemic stroke, combinations of anticoagulants and antiplatelet agents are utilised. However, despite adequate anticoagulation, APS patients may suffer recurrent life-threatening thrombotic events. A 10-year prospective study of 1000 patients with APS reported that 25% suffered from further thromboses despite antithrombotic therapy and were the most common cause of death (36.5% of total deaths over the 10-year period, 9.3% of the entire cohort) [14]. Importantly, warfarin can be poorly effective in triple aPL-positive patients and is associated with a ~30% risk of recurrence within 10 years despite otherwise adequate anticoagulation [5]. Direct oral anticoagulants such as rivaroxaban or apixaban are not as effective as warfarin, especially in patients with triple aPL positivity [15]. The management of recurrence by increasing the intensity of anticoagulation is limited by the risk of major bleeding including life-threatening or life-changing intracerebral haemorrhage (ICH).

**Figure 1:**
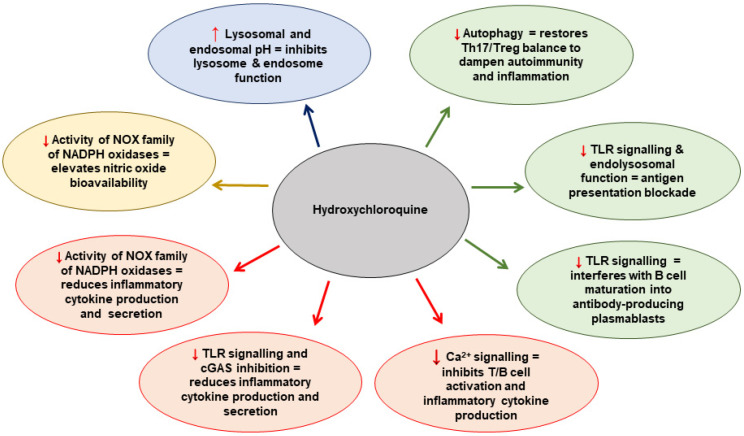
The proposed mechanisms that HCQ modulates at the cellular level. Adapted from Nirk et al. 2020 [17]. NADPH = nicotinamide adenine dinucleotide phosphate hydrogen; NOX = NADPH oxidase; TLR = Toll-like receptor; cGAS = cyclic GMP–AMP synthase; Th17 = T-helper cell 17.

The antimalarial drug hydroxychloroquine (HCQ) has been used for many years for the treatment of inflammatory rheumatic diseases, especially SLE and RA. HCQ is cheap and easily available. It has complex pleiotropic mechanisms of action [16] upon multiple cell types, collectively supporting the hypothesis that HCQ may act as a successful adjunctive treatment in the prevention of recurrent thrombosis or pregnancy complications. The proposed mechanisms that HCQ modulates at the cellular level are summarised in Figure 1 [17]. In this review, we discuss the role of HCQ as an immunomodulatory, antithrombotic and antiplatelet agent and its potential benefits for patients with thrombotic and obstetric APS.

## 2. Hydroxychloroquine in Thrombotic APS

The antithrombotic and immunomodulatory properties of HCQ were demonstrated many years ago in a thrombotic APS mouse model. Edwards et al. showed that HCQ significantly diminished both the thrombus size and duration of thrombus formation in mice injected with aPL [18]. More recently, Miranda et al. showed that HCQ reduced TF expression in arterial tissue isolated from thrombotic APS mice, as well as reduced the plasma levels of soluble cell adhesion molecules E-selectin and vascular cell adhesion molecule (VCAM-1). HCQ also improved endothelial nitric oxide synthase (eNOS) mRNA and protein expression, suggesting that it may stimulate the posttranscriptional expression of eNOS in the mouse model [19]. eNOS regulates NO production which, in turn, supports the endothelial antithrombotic function by inhibiting platelet activation and promoting vasodilation [20]. Interestingly, in a separate in vivo model, anti-β_2_GPI were reported to promote thrombosis by downregulation of eNOS and reducing generation of NO. These results were replicated in an in vitro system using cultured human EC [21].In a separate thrombotic APS mouse model, Urbanski et al. demonstrated that a single injection of monoclonal anti-β_2_GPIinduced arterial resistance that lasted up to three weeks; HCQ improved endothelium-dependent arterial dilation, which was mediated by an increase in eNOS [22].

Numerous other APS-related in vitro studies reported beneficial effects of HCQ upon the endothelium and other cell types. For example, HCQ reduced endothelial and monocyte TF expression [23], upregulated endothelial TM expression [19] and protected from aPL-mediated disruption of the endothelial annexin V anticoagulant shield [24]. In patients, HCQ treatment reduced soluble plasma TF [25]. Furthermore, Dong et al. showed that HCQ can reverse aPL-inhibited angiogenesis and endothelial migration [26]. In SLE, several lines of in vitro and in vivo evidence suggest that a significant benefit of HCQ may also arise from its effects on the endothelium, as seen in murine SLE models where early treatment with HCQ prevented the development of endothelial dysfunction [27]. HCQ has been shown to affect the antigen–antibody interaction (formation of immunocomplexes), an essential biological step that facilitates aPL-induced pathology. In a study using ellipsometry and atomic force microscopy (AFM), Rand et al. demonstrated HCQ at ≥1 μg/mL directly affected the formation of IgG aPL–β_2_GPI complexes on phospholipid bilayers. The incubation of IgG aPL–β_2_GPI with HCQ significantly reduced the binding of complexes to phospholipids compared with complexes incubated with the control buffer (no HCQ). This effect was completely reversed when the HCQ–protein solutions were dialyzed against the buffer (0.01 M HEPES, 0.14 M NaCl 0.05%) [28].

**Figure 2:**
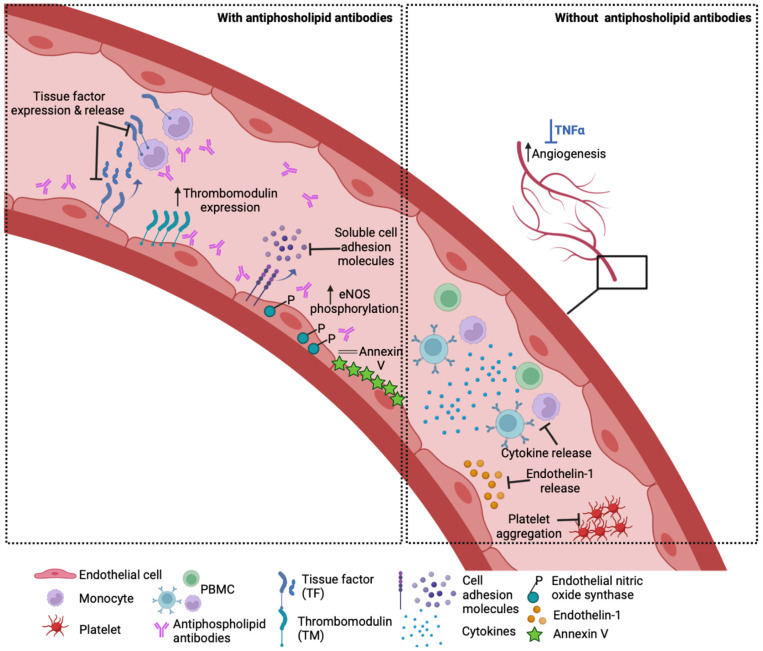
How HCQ may benefit the vasculature. Left panel: In the presence of antiphospholipid antibodies, HCQ has been shown to reduce endothelial and monocyte tissue factor expression in vitro and lower soluble TF levels in patients. Other in vitro and in vivo studies demonstrate that HCQ reverses the aPL-induced increase in soluble cell adhesion molecules, reduction in thrombomodulin expression and eNOS phosphorylation, as well as disruption of the anticoagulant annexin V shield. Right panel: HCQ ameliorates TNF-a induced endothelin-1 release by endothelial cells and prevents impaired angiogenesis mediated by TNF-a in vitro. HCQ also lowers the cytokine production from stimulated healthy peripheral blood mononuclear cells (PBMC) and agonist-stimulated platelet aggregation.

Outside the context of APS, HCQ was shown to block the release of the potent vasoconstrictor endothelin-1 and restore impaired angiogenesis in vitro in TNF-α-treated EC [29]. HCQ reduced the production of inflammatory cytokines such as TNF-α, IFN-γ and IL-6 from peripheral blood mononuclear cells [30,31]; upregulated IL-10 [32] and reduced platelet aggregation [33]. HCQ also targets lysosomal activity, autophagy, membrane stability and alters signalling pathways and transcriptional activity [16,34]. However, its exact mechanism of action and its cellular binding site are still not well understood, and its effects may vary according to the type of vascular endothelium examined (venous, arterial or microvascular). Experimental HUVEC models of inflammation showed that TNF-α-induced endothelial–leukocyte adhesion and the expression of endothelial ICAM-1 and VCAM-1 were profoundly reduced by HCQ treatment [29,35]. In an ex vivo vasomotion model, HCQ induced the relaxation of rat aortic rings in a concentration-dependent manner that was mediated by endothelium-derived NO [36]. In patients with chronic renal failure, HCQ caused a significant reduction in vascular endothelial dysfunction and improvement in vascular elasticity and flow with preservation of the blood vessel wall thickness [37]. The potential effects of HCQ on the vasculature are summarised in Figure 2.

The European Medicines Agency has licenced the use of HCQ for the treatment of APS as an orphan medicinal product in patients with refractory or recurrent thrombosis despite adequate anticoagulation [38]. Importantly, the risk of bleeding associated with HCQ is extremely low. HCQ therefore appears an excellent candidate as an adjunct to anticoagulation. In a small pilot open label randomised prospective study, 50 patients with primary APS were allocated in a 1:1 ratio to receive HCQ and anticoagulation with or without antiplatelet treatment vs. anticoagulation with or without antiplatelet treatment (no HCQ) [39]. In this study, at a 2.6-year follow-up, patients who received HCQ had lower rates of recurrent thrombosis (0.001 vs. 0.007, log-rank *p* = 0.048). However, following the adjustment for age, gender, vascular risk factors, presence of triple-positive aPL, previous history of recurrent thrombosis and time in therapeutic range of anticoagulation with VKAs, there was no effect of HCQ towards reducing the risk of recurrent thrombosis. This could be due to a lack of statistical power due to the small number of patients. Additionally, no adverse events were reported related to HCQ. Interestingly, HCQ reduced IgG aCL and both IgG and IgM anti-β_2_GPItitres [39]. Prior to the above study, a small prospective nonrandomised study included 40 patients allocated to receive HCQ (400 mg daily) with VKA (target INR 2.5) or VKA alone (*n* = 20 patients per treatment group) [40]. Over a follow-up period of 6–36 months, six patients developed recurrent thrombosis in the VKA alone group, whilst none had recurrence in the HCQ group. In contrast to the aforementioned randomised pilot study, HCQ had no effect on the aPL level. Importantly, there was again no difference in the bleeding events in the two groups [40]. Another study showed that treatment with HCQ reduced the aPL levels in patients with primary APS [41]. Evidence supporting the vasculoprotective effects of HCQ was also reported in a large case–control study of 10,268 cases and 29,969 controls that evaluated HCQ use and the occurrence of cardiovascular events among patients with SLE or RA. The study found a lower risk of overall cardiovascular events, lower risk of venous thromboembolism and a trend towards lower risks for myocardial infarction and ischemic stroke associated with the current HCQ use [42]. Additionally, a systematic review and meta-analysis of HCQ in rheumatic disease showed a significant reduction in the risk of cardiovascular events (pooled risk ratio, RR, 0.72, 95% CI 0.56–0.94, *p* = 0.013) [43].

## 3. Hydroxychloroquine in Obstetric APS

Placental thrombosis, inflammation and complement activation all play major roles in the pathogenesis of obstetric APS [44]. Heparin and low-dose aspirin (LDA) are the treatment of choice for obstetric complications. This treatment regime is based on several clinical studies and meta-analyses on the use of heparin and LDA in obstetric APS [45]. Low molecular weight heparin (LMWH) is the preferred heparin and has been the standard of care due to its lower risk of heparin-induced thrombocytopenia (HIT) and osteopenia and the relatively easy subcutaneous administration compared to unfractionated heparin (UFH). With standard treatment, approximately 70% of pregnant women with APS have successful pregnancy outcomes [46]. However, for the remaining 20–30% of women with obstetric APS, there are limited therapeutic options and no guidelines for those who do not respond to heparin and LDA [46,47,48]. Several adjunctive treatments have been used in women with refractory obstetric APS with variable success [49]. These include steroids, intravenous immunoglobulin, plasma exchange and HCQ [49]. Retrospective studies reported better outcomes with patients with obstetric APS treated with HCQ in addition to the standard treatment [50]. Sciascia et al. reported that HCQ treatment was associated with a higher rate of live births (67% vs. 57%; *p* = 0.05) and a lower prevalence of aPL-related pregnancy morbidity (47% vs. 63%; *p* = 0.004) [51]. Gerde et al. reported on a retrospective study assessing the effects of HCQ in achieving successful pregnancy outcomes [52]. In this study, pregnancy outcomes (live birth rates as the primary outcome; early and late pregnancy loss and placental mediated complications as secondary outcomes) in 87 women with refractory primary obstetric APS and no other autoimmune disease who received 400 mg HCQ with 60 mg LMWH and LDA were compared with women who received 60 mg LMWH and LDA. Both the primary and secondary outcomes were significantly better in women treated with HCQ (97.1% (67/69) vs. 62.5% (20/32); *p* < 0.001). Overall, the pregnancy complications were lower in women treated with HCQ compared to the standard treatment alone (8.7% (6/69) vs. 37.5% (12/32); *p* < 0.001) [52]. Currently, three prospective clinical trials (HYPATIA: A prospective randomised controlled trial of HYdroxychloroquine to improve Pregnancy outcome in women with AnTIphospholipid Antibodies; HIBISCUS: Hydroxychloroquine for the secondary prevention of thrombotic and obstetrical events in primary antiphospholipid syndrome; HYDROSAPL: HYDROxychloroquine in Syndrome Primary AntiPhospholipid) are assessing the safety and efficacy of HCQ in obstetric APS in combination with the standard treatment. These studies will provide us with more definitive evidence of the use of HCQ in the prevention of obstetric complications.

Similarly, the clinical utility of HCQ has been demonstrated in in vivo models of obstetric APS, preventing foetal death and placental metabolic changes [53]. HCQ protected not only from aPL-mediated placental insufficiency but also abnormal foetal brain development [53]. In the same study, the authors showed that HCQ prevented complement activation both in vivo and in vitro. APS mice had lower C5a levels following treatment with HCQ, and this was observed in patients with APS as well [53]. In cultured human extravillous trophoblast cells, Liu et al. demonstrated that aPL reduced the ability of normal trophoblast cells to invade, migrate and form tubules, which was partially reversed by HCQ [54]. Furthermore, in an obstetric APS mouse model, HCQ was able to reduce pregnancy complications and prevent foetal death [54]. Wu et al. showed that HCQ reversed the effects of aPL on primary syncytiotrophoblasts generated from cytotrophoblasts in vitro by significantly reducing IgG binding and restoring annexin A5 expression [55]. Using the human placental BeWo cell line as a model for trophoblast fusion and differentiation, Marchetti et al. showed that anti-β_2_GPI decrease trophoblastic differentiation via Toll-like receptor 4 (TLR4). HCQ reversed this phenotype and reduced TLR4 expression levels [56]. Finally, in a human first-trimester trophoblast cell line (SVneo-transformed HTR8 cells), HCQ was able to partially reverse the aPL-mediated inhibition of cell migration despite the observation that HCQ treatment increased the secretion of promigratory IL-6 by trophoblasts [57].

## 4. Hydroxychloroquine for Asymptomatic aPL Carriers

Individuals with aPL but no history of thrombosis (aPL carriers), although usually asymptomatic at the time of detection, are also at increased risk of developing thrombosis and thereby join the APS group. A multicentre prospective observational study of individuals with a high-risk aPL profile (triple-positive) demonstrated a thromboembolism rate of 5.3% per year, with a cumulative incidence of 37.1% after 10 years (95% CI, 19.9–54.3%) [58]. Another study of asymptomatic carriers reported the annual first thrombosis rate of 2.3% per patient/year during a median of 13 years and noted the risk to be three-fold higher in triple aPL-positive carriers (OR 3.38 (95% CI: 1.24–9.22)) [58]. The identification of this high-risk group raises the question of whether they should be offered primary prophylaxis. However, anticoagulation carries a high risk of bleeding and no evidence to support its implementation in this setting. A recent Preventive Services Task Force (USPSTF) report suggests that primary prophylaxis with aspirin should only rarely be used for the prevention of myocardial infarction (MI) in the general population [59]. Thus, an anti-inflammatory approach may be preferable for this group. In a cross-sectional study of aPL-positive patients with connective tissue disease and APS, a logistic regression analysis demonstrated the reduced risk of a thrombotic event by taking LDA and/or HCQ [60]. Another pilot randomised prospective study of 65 patients investigated the impact of HCQ on thrombosis development and aPL titres in both APS patients and aPL carriers. The results of this trial showed that the use of HCQ plus the standard care was associated with a lower incidence of thrombosis during a 2.6-year follow-up [60]. In a small, randomised control study with 20 aPL carriers where 9/20 were randomised to HCQ, this study was prematurely terminated due to logistic reasons. However, over a 1.7-year follow-up period, no patients developed thrombosis or a serious adverse event [61].

## 5. Conclusions

HCQ has pleotropic effects on multiple cell types. The drug’s effects on EC, immune cells and platelets in particular, modulate inflammation and the thrombotic risk. The potential benefits of HCQ in reducing the risk of thrombosis has been demonstrated in thrombotic APS and asymptomatic carriers of aPL from in vivo, in vitro and clinical studies. Importantly, HCQ is not associated with an increased risk of bleeding when used in combination with an anticoagulation or in isolation. HCQ’s biological effects may also contribute to protection in obstetric APS given the central role of the vasculature during foetal/placental development. However, there remains a need for more robust evidence on HCQ’s mechanisms of action in reducing thrombosis and obstetric complications to definitively demonstrate the clinical benefits and promote the establishment of multicentre clinical trials assessing the development of APS and the risk of thrombotic or obstetric recurrence.

## Data Availability

Not applicable.

## Abbreviations

anti-β_2_GPI	anti-β_2_-glycoprotein I
aCL	Anticardiolipin antibodies
Anti-PS/PT	anti-phosphatidylserine/prothrombin antibodies
APS	Antiphospholipid syndrome
aPL	Antiphospholipid antibodies
EC	Endothelial cells
EPCR	endothelial protein C receptor
HCQ	hydroxychloroquine
ICH	intracerebral haemorrhage
LA	lupus anticoagulant
LDA	low dose aspirin
LMWH	Low molecular weight heparin (LMWH
NO	Nitric oxide
eNOS	Nitric oxide synthase
PAI-I	Plasminogen activator inhibitor I
RA	Rheumatoid arthritis
SLE	systemic lupus erythematosus
TM	Thrombomodulin
TF	Tissue factor
TFPI	Tissue factor pathway inhibitor
tPA	Tissue-plasminogen activator
TLR4	Toll like receptor 4
UFH	unfractionated heparin
USPSTF	US Preventive Services Task Force
VKA	vitamin K antagonists
VWF	von Willebrand factor
